# Ultrasensitive detection of oncogenic human papillomavirus in oropharyngeal tissue swabs

**DOI:** 10.1186/s40463-016-0177-8

**Published:** 2017-01-14

**Authors:** Andre Isaac, Morris Kostiuk, Han Zhang, Cameron Lindsay, Fawaz Makki, Daniel A. O’Connell, Jeffrey R. Harris, David W. J. Cote, Hadi Seikaly, Vincent L. Biron

**Affiliations:** 1 Division of Otolaryngology-Head and Neck Surgery, Department of Surgery, University of Alberta, 1E4.34 WMC, 8440 112 Street, Edmonton, AB T6G 2B7 Canada; 2 1E4 WMC, 8440 112 Street, Edmonton, AB T6G 2B7 Canada; 3 1E4.29 WMC 8440 112 Street, Edmonton, AB T6G 2A1 Canada; 4 1E4 WMC 8440 112 Street, Edmonton, AB T6G 2A1 Canada

**Keywords:** Head and neck cancer, HPV, Diagnostic study, p16, ddPCR, E6 and E7

## Abstract

**Background:**

The incidence of oropharyngeal squamous cell carcinoma (OPSCC) caused by oncogenic human papillomavirus (HPV) is rising worldwide. HPV-OPSCC is commonly diagnosed by RT-qPCR of HPV E6 and E7 oncoproteins or by p16 immunohistochemistry (IHC). Droplet digital PCR (ddPCR) has been recently reported as an ultra-sensitive and highly precise method of nucleic acid quantification for biomarker analysis. To validate the use of a minimally invasive assay for detection of oncogenic HPV based on oropharyngeal swabs using ddPCR. Secondary objectives were to compare the accuracy of ddPCR swabs to fresh tissue p16 IHC and RT-qPCR, and to compare the cost of ddPCR with p16 IHC.

**Methods:**

We prospectively included patients with p16^+^ oral cavity/oropharyngeal cancer (OC/OPSCC), and two control groups: p16^−^ OC/OPSCC patients, and healthy controls undergoing tonsillectomy. All underwent an oropharyngeal swab with ddPCR for quantitative detection of E6 and E7 mRNA. Surgical specimens had p16 IHC performed. Agreement between ddPCR and p16 IHC was determined for patients with p16 positive and negative OC/OPSCC as well as for healthy control patients. The sensitivity and specificity of ddPCR of oropharyngeal swabs were calculated against p16 IHC for OPSCC.

**Results:**

122 patients were included: 36 patients with p16^+^OPSCC, 16 patients with p16^−^OPSCC, 4 patients with p16^+^OCSCC, 41 patients with p16^−^OCSCC, and 25 healthy controls. The sensitivity and specificity of ddPCR of oropharyngeal swabs against p16 IHC were 92 and 98% respectively, using 20–50 times less RNA than that required for conventional RT-qPCR. Overall agreement between ddPCR of tissue swabs and p16 of tumor tissue was high at ĸ = 0.826 [0.662-0.989].

**Conclusion:**

Oropharyngeal swabs analyzed by ddPCR is a quantitative, rapid, and effective method for minimally invasive oncogenic HPV detection. This assay represents the most sensitive and accurate mode of HPV detection in OPSCC without a tissue biopsy in the available literature.

**Electronic supplementary material:**

The online version of this article (doi:10.1186/s40463-016-0177-8) contains supplementary material, which is available to authorized users.

## Background

Head and neck squamous cell carcinoma (HNSCC) is now the fifth most common malignancy worldwide [1]. The incidence of oropharyngeal squamous cell carcinoma (OPSCC), a subsite of HNSCC, is rapidly increasing in North America [2]. Although traditional risk factors for HNSCC include smoking and alcohol use, the rising incidence of OPSCC has been attributed largely to the Human Papillomavirus (HPV) [3–6]. Determining HPV positivity is of critical importance in the diagnosis and management of OPSCC, as HPV positive tumours have unique pathologic and clinical characteristics that have implications for prognosis and treatment decisions [3, 7–10].

The gold standard for determining HPV status in OPSCC is demonstration of oncogenic HPV DNA in fresh tissue using real-time quantitative polymerase chain reaction (RT-qPCR) [1, 11, 12]. Due to the high cost and specialized equipment required for this method, most centers have adopted p16 immunohistochemistry (p16 IHC) as the preferred method of oncogenic HPV detection, which has become the clinical standard. [13–17] However, p16 IHC is an imperfect surrogate marker for HPV-associated OPSCC. HPV infection is carcinogenic through expression of oncogenic proteins E6 and E7 which cause multiple genetic and metabolic effects within the cell, the most important of which is degradation of tumor suppressor genes including p53 and Rb [18]. By a separate pathway, this results in downstream overexpression of p16. Because p16 overexpression can occur through HPV-independent pathways, p16 IHC can lead to false positives, which could result in erroneous de-intensification of treatment. Although RT-PCR of E6 and E7 can circumvent the limitations of p16 IHC, it also requires adequate nucleic acid sample generally only attainable from tissue biopsy.

Droplet digital polymerase chain reaction (ddPCR) is a relatively novel technique with potential applications for HPV detection in OPSCC. ddPCR is the most accurate and sensitive mode of measuring target nucleic acids quantitatively in the available literature [19]. ddPCR involves partitioning a single nucleic acid sample in up to 20,000 discrete water-in-oil droplets and performing PCR analysis on each droplet independently, with the results reported digitally and quantitatively. This technique quantifies the absolute amount of target nucleic acid present with greater precision and reproducibility than RT-qPCR [20–23]. ddPCR has been used to quantify gene expression with extremely low copy number [24, 25], and has demonstrated better diagnostic performance in biomarker analysis than other molecular techniques. [22, 26, 27] One study made use of ddPCR for detection of oncogenic E6/E7 mRNA in fresh tissue OPSCC specimens and found 100% sensitivity compared to p16 IHC using target RNA 20–50 times lower than reported for RT-qPCR [28].

Because of the accuracy and ultra-sensitivity of ddPCR for identification of oncogenic HPV E6/E7 mRNA, we hypothesized that ddPCR can be used for detection of oncogenic HPV in OPSCC using oral/oropharyngeal swabs as opposed to fresh tissue. The ability to detect oncogenic HPV without a tissue biopsy would have important implications for diagnosis, post-treatment surveillance, and screening of patients with OPSCC.

The purpose of this study was to validate the use of a novel minimally-invasive assay for detection of oncogenic HPV based on oral/oropharyngeal swabs using ddPCR. Our secondary objectives were to compare the accuracy of ddPCR swabs to fresh tissue p16 IHC, and to report the cost of ddPCR.

## Methods

This was a single-center prospective cohort validation study at a tertiary care Otolaryngology-Head and Neck Surgery referral center in Edmonton, AB, Canada. Health research ethics board approval was obtained from the University of Alberta prior to commencement of the study (Pro00057994).

### Participants

Participants were recruited at initial presentation to the University of Alberta Otolaryngology-Head and Neck Surgery Clinic between February 2015 and March 2016. Adult patients with biopsy-confirmed oral cavity or oropharyngeal squamous cell carcinoma (OC/OPSCC) were identified. Patients who had previous treatment for HNSCC, those that were unable to undergo an oropharyngeal swab, patients for which pathology report or p16 IHC was unavailable, patients with unknown primary tumours, and improperly processed samples were excluded. The reason for including OCSCC patients in addition to OPSCC was to be able to compare ddPCR and p16 in a subgroup of patients for which p16 is an especially poor marker of HPV infection, in order to determine the enhanced specificity of ddPCR for HPV oncogenesis.

The control group was recruited from patients who were being consented for tonsillectomy for a benign indication (ex: recurrent tonsillitis or obstructive sleep apnea). Patients were excluded from the control group if they had a previous history of HNSCC.

### Oral/oropharyngeal swabs

Each participant underwent an oral/oropharyngeal swab using a 10 cm cotton-tip applicator by a staff Otolaryngologist (VB, HS, JH, or DO). Two swabs were performed on each patient. For patients with a clinically evident oral/oropharyngeal tumour, one swab was taken from the tumour, and the second swab was taken from the oropharynx (a single swab that was brushed against the tonsils, base of tongue, soft palate, and posterior pharyngeal wall subsites). For control patients, two swabs were taken both from the oropharyngeal subsites listed above (each swab was brushed against all of the subsites listed above). The swab tips were immediately placed in 3 mL of RNAlater (Ambion-Thermo Fisher Scientific, Waltham, MA, USA) and stored at room temperature (RT) for up to 24 h, then at 4 °C for up to 7 days prior to RNA extraction.

### Tissue pathology and p16 IHC

Each patient with an oral/oropharyngeal tumour underwent pan-endoscopy with biopsy of the tumour as per standard clinical practice. Pathology was reported by a head and neck pathologist at the University of Alberta to confirm the diagnosis of SCC. p16 IHC was performed on representative 4 μm sections cut from formalin-fixed, paraffin-embedded tissue blocks using a monoclonal antibody to p16, as per established guidelines [29]. For control patients, tonsil specimens were sent for pathologic analysis at the time of tonsillectomy and were interpreted by an anatomic pathologist at the University of Alberta to confirm the diagnosis of non-malignant tonsil tissue. P16 was not performed if the tissue was deemed benign, since the significance of p16 in the absence of carcinoma is unclear. Instead, these patients were used as negative controls.

### RNA extractions and cDNA synthesis

RNA was extracted from tumor tissue using the RNeasy Mini Kit (Qiagen, Germantown, MD, USA) following the manufacturer’s protocol. Salivary swab samples were vortexed in 15 mL conical tubes containing 3 mL of RNAlater and centrifuged at 3000 x g for 10 min. The RNAlater was then aspirated and cell pellet was re-suspended in 350 μL of buffer RLT containing 40 mM DTT. RNA was eluted from the mini column with 35 μL of RNAse free water. RNA concentration was quantified using the Qubit RNA HS assay kit on a Qubit 2.0 fluorometer.

Extracted RNA (100–200 ng) was used to synthesize cDNA using the iScriptTM Reverse Transcription Supermix for RT-qPCR (BIO-RAD) as per the manufacturer’s protocol. Following the reaction the cDNA was diluted with 0.125 mM EDTA pH 8.0 to 0.5 ng/μl and either stored at −20 °C or used directly for ddPCR*.*

### Droplet digital PCR

All ddPCR reactions were performed by MK, who was blinded to the patient group, pathology, and p16 IHC status of participant samples. ddPCR was carried out using the ddPCRTM Supermix for Probes (No dUTP) (BIO-RAD, Mississauga, ON, CAN), the QX200TM Droplet Generator (catalog #186-4002 BIO-RAD), the QX200 Droplet Reader (catalog #186-4003 BIO-RAD) the C1000 TouchTM Thermal Cycler (catalog #185-1197 BIO-RAD) and the PX1TM PCR Plate Sealer (catalog #181-4000 BIO-RAD) as per manufacturer’s instructions. Reactions were set up following the manufacturer’s protocols using 12 μl/reaction of 2x ddPCR Supermix for Probes (No dUTP), 1.2 μL/reaction of 20x target primers/probe (FAM or HEX, BIO-RAD), 1.2 μL/reaction 20x reference primers/probe (FAM or HEX, BIO-RAD), 2.4 μL cDNA (at 0.5 ng/μl) and 7.2 μl H2O. Human EEF2 primers/probes (BIO-RAD) were used as an internal reference standard and indirect indicator of nucleic acid stability in participant samples. HPV E6 and E7 ddPCR detection was performed using the following primer/probe sequences generated by BIO-RAD [30], adapting primer sequences from HPV E6: forward sequence, 5′-TCAGGACCCACAGGAGCG-3′, reverse sequence, 5′-CCTCACGTCGCAGTAACTGTTG-3′, probe (FAM-labeled) sequence, 5′-CAGAAAGTTACCACAGTTATGCACAGAGCT-3′. HPV E7: forward sequence, 5′-CCGGACAGAGCCCATTACAA -3′, reverse sequence, 5′-CGAATGTCTACGTGTGTGCTTTG -3′, probe (HEX-labeled) sequence, 5′-CGCACAACCGAAGCGTAGAGTCACACT -3′. Reactions were set up in a 96 well plate, mixed using a Mixmate Vortex Shaker (Eppendorf, Mississauga, ON, CAN) and 20 μl of the reaction mixture was transferred to DG8TM Cartridge for QX200/QX100 Droplet Generator (catalog #186-4008 BIO-RAD) followed by 70 μl of Droplet Generation Oil for Probes (catalog #186-3005 BIO-RAD) into the oil wells, according to the QX200 Droplet Generator Instruction Manual (#10031907 BIO-RAD). Following droplet generation, 40 μL of the reaction was transferred to wells of a 96 well plate. The plates were sealed and the reactions were carried out in the thermocycler using the following parameters: Step 1) 95 °C for 10 min, Step 2) 94 °C for 30 s and 60 °C for 1 min (Step 2 repeat 39 times for a total of 40), Step 3) 98 °C for 10 min and Step 4) 4 °C infinite hold. All steps had a ramp rate of 3 °C/s. Following thermocycling the reactions were read in the QX200 Droplet Reader and the RNA targets were quantified using the QuantaSoftTM Software (BIO-RAD). HPV E6 and E7 positivity was determined using automated cutoff values relative to control as previously described [28]. Samples with > 2 droplets in the positive range for E6 or E7 were considered positive. Samples with < 20 positive droplets were re-analyzed to ensure these low copy number samples were not due to cross-contamination.

### Validation of ddPCR sensitivity against RT-qPCR

As previously shown in other studies, we confirmed the enhanced sensitivity of ddPCR over RT-qPCR using decreasing concentrations of target RNA for EEF2 and E7 (see Additional file 1: Table S1). As recommended by the manufacturer (BioRAD), the same EEF2 and E7 primer/probe sets used in ddPCR were used for qRT-PCR with optimized annealing temperatures. RT-qPCR was performed as follows: RNA (100 ng) was used to synthesize cDNA using the iScriptTM Reverse Transcription Supermix for RT-qPCR (BIO-RAD) as per the manufacturer’s protocol in a 20 ul reaction. The resulting cDNA was diluted to 1.25 ng/ul, 0.125 ng/ul and 0.0125 ng/ul with water and these dilutions were used to load 10 ng, 1.0 ng and 0.1 ng respectively into reaction wells for either the qPCR or ddPCR. 20 ul final qPCR reactions were set up following the manufacturer’s protocols using 10 ul/reaction of 2x iTaqTM Universal Probes Supermix (BIO-RAD), 1 ul/reaction of 20x target primers/probe (FAM or HEX, BIO-RAD), 1 ul/reaction 20x reference primers/probe (FAM or HEX, BIO-RAD) and 8 ul of cDNA. Reactions were carried out in the CFX96 Touch™ Real-Time PCR Detection System using the “Prime PCR” Manufacturer’s program with the following parameters: Step 1) 95 ° C for 2 min, Step 2) 95 ° C for 5 s and 60 ° C for 30 s (Step 2 repeat 39 times for a total of 40), Step 3) 95 ° C for 5 s.

### Data analysis

Descriptive statistics were performed to determine the proportion of patients recruited with p16 positive vs. negative OC/OPSCC. Agreement between ddPCR and p16 IHC was determined for patients with p16 positive and negative OC/OPSCC as well as for healthy control patients. The sensitivity and specificity of ddPCR of oropharyngeal swabs were calculated against p16 IHC for OPSCC.

## Results

122 patients met the inclusion and exclusion criteria and were prospectively enrolled in the study. These patients are summarized in Table 1. There were 36 patients with p16 positive OPSCC (p16^+^OPSCC), 16 patients with p16 negative OPSCC (p16^−^OPSCC), 4 patients with p16^+^OCSCC, and 41 patients with p16^−^OCSCC. 25 patients were healthy controls. The mean concentration of RNA obtained from oral/oropharyngeal swabs was 5.31 μg/mL (range 2.2-12.1 μg/mL). The minimum target RNA required per reaction was ≤1 ng.

**Table 1:** Patient demographics and diagnoses

Patient Variable		Number of patients (%) *n* = 122
Mean Age ± SD		57 ± 15 years
Males		74 (67%)
Oropharyngeal SCC	p16 +	36 (30%)
	p16 -	16 (13%)
Oral Cavity SCC	p16 +	4 (3%)
	p16 -	41 (34%)
T stage (SCC patients only)	T1	19 (20%)
	T2	24 (25%)
	T3	15 (15%)
	T4	39 (40%)
Smoking History (all patients)		46 (38%)
Normal tonsil		25 (20%)

*SCC* squamous cell carcinoma

33/36 (92%) of patients with p16^+^OPSCC tested positive for E6/E7 by ddPCR (Table 2). One patient with p16^−^OPSCC tested positive for E6/E7 by ddPCR. The sensitivity of ddPCR of oropharyngeal swabs against fresh tissue p16 IHC was 92%. All 4 patients with p16^+^OCSCC tested negative for E6/E7 by ddPCR, as did all 41 patients with p16^−^OCSCC (Table 3). All 25 healthy control patients tested negative for E6/E7 by ddPCR, for an overall specificity of 98% for OPSCC. The agreement between ddPCR and p16 via unweighted kohen’s kappa was ĸ = 0.826 [0.662-0.989]

**Table 2:** p16 IHC and ddPCR E6/E7 Results in Patients with OPSCC

	p16 +	p16 -	Totals
ddPCR +	33	1	34
ddPCR -	3	15	18
Totals	36	16	

*IHC* immunohistochemistry, *ddPCR* droplet digital polymerase chain reaction, *OPSCC* oropharyngeal squamous cell carcinoma

**Table 3:** p16 IHC and ddPCR E6/E7 Results in Patients with OCSCC

	p16 +	p16 -	Totals
ddPCR +	0	0	0
ddPCR -	4	41	45
Totals	4	41	

*IHC* immunohistochemistry, *ddPCR* droplet digital polymerase chain reaction, *OCSCC* oral cavity squamous cell carcinoma

The cost of p16 IHC across the province of Alberta is $31.10/slide, with a minimum of 2 slides required per patient (≥ $62.10/patient). In comparison, the total cost of HPV E6/E7 ddPCR including technical labour was estimated to be $20.45 per patient sample.

## Discussion

The incidence of HPV-related OPSCC is rapidly growing and poses challenges for diagnosis and management. Accurate determination of HPV status in patients with OPSCC is therefore critical. Performing HPV testing using a method that is cost effective and minimally invasive while maintaining adequate sensitivity has many potential applications. The method presented here is to our knowledge the most sensitive method of diagnosing oncogenic HPV mRNA without a tissue biopsy reported to date. Every swab performed in this study yielded adequate RNA for amplification, with 1 ng of RNA/reaction required for robust ddPCR results. This is an order of magnitude less than the 20–50 ng/reaction of RNA that is normally required for RT-qPCR [30–33]. This is also to our knowledge the first study to compare non-invasive oncogenic HPV detection with the clinical reference standard of p16 IHC. In addition to the greater specificity of using an HPV-specific assay, our cost analysis demonstrates a significant cost-savings of ddPCR over p16 IHC for determining HPV status in OPSCC.

Overall our results demonstrated excellent sensitivity in detecting oncogenic HPV in oropharyngeal swabs without a biopsy, as 92% of p16^+^OPSCC tested positive by ddPCR. The three patients with p16^+^OPSCC that tested negative for E6/E7 by ddPCR may in fact highlight the limitations of p16 IHC as a surrogate marker for HPV, as these three patients were older and had significant smoking and alcohol use histories, and thus may have had disease that was unrelated to HPV. While studies have suggested that p16 is itself an important prognostic marker independent of HPV infection in OPSCC [13, 34], others have questioned the prognostic utility of p16 in HPV negative tumors [35], and have demonstrated improved prognostication when HPV-specific tests are used [36]. Robinson et al. argued that HPV-specific testing remains essential in OPSCC regardless of p16 status [37].

Our results also demonstrated a high degree of accuracy in determining HPV status in OCSCC, as all 4 patients with p16^+^OCSCC tested negative for E6/E7 by ddPCR. Other studies have shown that non-OPSCC that are p16 positive are usually unrelated to HPV, and that p16 positivity does not purport a better prognosis in non-OPSCC, and may in fact yield a worse prognosis in these patients [38–41]. This adds to the clinical utility of using HPV-specific ddPCR as opposed to or in addition to p16 IHC in both OPSCC and non-OPSCC.

The fact that the assay we have described yielded excellent sensitivity without the need for a tissue biopsy has several important implications, the most immediately relevant of which may be for post-treatment surveillance. One recent study claimed to be able to predict recurrence of OPSCC earlier using salivary rinses for detection of oncogenic HPV [42], however this assay was limited by a lack of sensitivity due to the large amount of RNA required for RT-qPCR. A similarly designed study by Chuang et al. reported a sensitivity of 50% in predicting recurrent OPSCC using oral rinses for HPV DNA using RT-qPCR, although this study only included 4 patients with recurrent OPSCC [43]. Ahn et al. also used oral rinses for HPV DNA to predict recurrent OPSCC and found that when combined with plasma RT-qPCR, a sensitivity of almost 70% could be achieved; however, the pre-treatment sensitivity of oral rinses was only 53% [30]. With the significantly improved sensitivity of the novel assay we have described, post-treatment surveillance may be more effective using regular oropharyngeal swabs to detect E6/E7 via ddPCR. The use of ddPCR for post-treatment surveillance needs further research however before this can be recommended or widely utilized.

Other studies that have attempted to determine HPV status using oral rinses and various PCR-based assays in a diagnostic fashion as opposed to post-treatment surveillance have reported similarly low sensitivities. Both Zhao et al. and Nordfors et al. compared RT-qPCR in oral rinses with tissue biopsies in patients with OPSCC and reported sensitivities of 30 and 68% respectively for HPV 16 DNA [44, 45]. With the improved sensitivity provided by our ddPCR-based assay, clinicians may be able to use the information diagnostically to make a diagnosis of HPV-related OPSCC sooner. This is particularly useful in settings where a biopsy may be difficult to obtain, or as part of the work-up for an unknown primary tumor. Moreover, with the rising prevalence of HPV-related OPSCC, our ddPCR assay may be a viable mode of screening high-risk patient groups for early detection or prevention of OPSCC in the future. Fahkry et al. attempted to validate a “cervical pap smear-equivalent” test for early detection of HPV-related OPSCC using a combination of oral rinses and tonsillar brush biopsies via RT-qPCR; however, they concluded that it was not feasible due to the lack of correlation between HPV DNA detection and cellular atypia [46]. The authors postulated that this was due to the difficulty in detecting HPV DNA that may be replicating deep within the tonsillar crypt epithelium, a problem that could potentially be solved by an ultra-sensitive ddPCR-based assay.

This study had limitations, which included a single center experience and a relatively small sample size especially for p16^+^OCSCC and p16^−^OPSCC. Our sensitivity and specificity data also need to be interpreted cautiously, as these were calculated against the results of p16 IHC, which is itself known to be a surrogate marker; however, our aim was to demonstrate the sensitivity of a swab-based assay against the mostly widely-used tissue-based assay for determining HPV status. We also only tested for HPV 16 as in other studies; however, HPV 16 is known to cause more than 95% of HPV-related OPSCC [46]. We plan to expand our assay to test for HPV 18 in addition to HPV 16.

## Conclusion

Oropharyngeal swabs analyzed by ddPCR is a quantitative, rapid, and cost-effective method for minimally invasive oncogenic HPV detection. This assay represents the most sensitive and accurate mode of HPV detection in OPSCC without a tissue biopsy in the available literature, and has several potential applications for both diagnosis and disease surveillance.

## Additional file

Additional file 1: Table S1. Sensitivity of ddPCR and RT-qPCR for detection of target EEF2 and E7 RNA in serially diluted samples. (DOC 82 kb)

## Abbreviations

ddPCR: Droplet digital polymerase chain reaction
HNSCC: Head and neck squamous cell carcinoma
HPV: Human papillomavirus
IHC: Immunohistochemistry
OCSCC: Oral cavity squamous cell carcinoma
OPSCC: Oropharyngeal squamous cell carcinoma
RT-qPCR: Real time quantitative polymerase chain reaction
