# Supplementary material for: Ultrasensitive detection of oncogenic human papillomavirus in oropharyngeal tissue swabs
Source: J Otolaryngol Head Neck Surg. 2017 Jan 14;46:5. doi: 10.1186/s40463-016-0177-8 (PMC5237494; doi:10.1186/s40463-016-0177-8)
Supplement: Supplementary file 1 [file 40463_2016_177_MOESM1_ESM.doc]

Supplemental Table A: Sensitivity of ddPCR and RT-qPCR for detection of target EEF2 and E7 RNA in serially diluted samples

Sample Number	Dilution (ng)	Target Expression Levels RT-qPCR (Cq)		Target Expression Levels ddPCR (Copies/20uL)	
		EEF2	E7	EEF2	E7
1	10	20.77	27.23	61400.0	5140
	1	24.03	30.40	6820.0	506
	0.1	27.04	34.14	676.0	56
2	10	19.85	21.70	123200	116000
	1	22.97	25.27	14140	12860
	0.1	26.55	28.61	1440	1398
3	10	22.72	0	12600	8.2
	1	26.57	0	1258	1.6
	0.1	32.86	0	158	0
4	10	21.47	25.20	40600	18520
	1	24.97	28.81	4600	2020
	0.1	27.65	32.11	452	206
5	10	24.26	36.71	6340	76
	1	28.02	0	640	3
	0.1	31.05	0 (39.61*)	70	0
6	10	21.31	26.32	37440	6760
	1	25.06	29.88	3800	752
	0.1	28.37	33.21	368	76
7	10	22.07	26.82	26160	5620
	1	25.36	30.19	2740	612
	0.1	29.03	33.61	272	96
8	10	19.98	29.39	88400	1840
	1	23.31	32.58	9020	168
	0.1	27.05	36.75	968	14.2

*False positive given high Cq.
